# Enhanced Production of Bryonolic Acid in *Trichosanthes cucumerina* L. (Thai Cultivar) Cell Cultures by Elicitors and Their Biological Activities

**DOI:** 10.3390/plants9060709

**Published:** 2020-06-02

**Authors:** Pornpatsorn Lertphadungkit, Jiraphong Suksiriworapong, Veena Satitpatipan, Supaart Sirikantaramas, Amaraporn Wongrakpanich, Somnuk Bunsupa

**Affiliations:** 1 Department of Pharmacognosy, Faculty of Pharmacy, Mahidol University, Bangkok 10400, Thailand; pornpatsorn.lep@student.mahidol.ac.th (P.L.); veena.nuk@mahidol.ac.th (V.S.); 2 Department of Pharmacy, Faculty of Pharmacy, Mahidol University, Bangkok 10400, Thailand; jiraphong.suk@mahidol.ac.th (J.S.); amaraporn.won@mahidol.ac.th (A.W.); 3 Molecular Crop Research Unit, Department of Biochemistry, Faculty of Science, Chulalongkorn University, Bangkok 10330, Thailand; Supaart.S@chula.ac.th

**Keywords:** bryonolic acid, callus, cell suspension, elicitors, *Trichosanthes cucumerina* L.

## Abstract

Bryonolic acid is a triterpenoid compound found in cucurbitaceous roots. Due to its biological activities, this compound gets more attention to improve production. Herein, we carried out efficient ways with high bryonolic acid productions from *Trichosanthes cucumerina* L., a Thai medicinal plant utilizing plant cell cultures. The results showed that calli (24.65 ± 1.97 mg/g dry weight) and cell suspensions (15.69 ± 0.78 mg/g dry weight) exhibited the highest bryonolic acid productions compared with natural roots (approximately 2 mg/g dry weight). In the presence of three elicitors (methyl jasmonate, yeast extract, and chitosan), cell suspensions treated with 1 mg/mL of chitosan for eight days led to higher bryonolic acid contents (23.56 ± 1.68 mg/g dry weight). Interestingly, cell culture and root extracts with high bryonolic acid contents resulted in significantly higher percent cell viabilities than those observed under control (1% *v/v* DMSO) treatment in Saos-2 and MCF-7 cells. The present study indicated that *T. cucumerina* L. cell cultures are alternative and efficient to produce the biologically important secondary metabolite.

## 1. Introduction

Medicinal plants have been used as major natural sources in drug development over recent decades. Owing to their activities, there are many reports that compounds isolated from plants have been used as modern drugs against many human diseases. As far as everybody knows, the chemical synthesis of valuable natural compounds is difficult because of the structural complexities, especially triterpenoids. For solving this problem with the aim of producing a large amount of natural compound, plant biotechnological knowledge, including plant cell cultures, have been developed. Bryonolic acid is an active triterpenoid compound with various interesting potentials. This compound exerts anti-allergic activity by inhibiting homologous passive cutaneous anaphylaxis and delayed hypersensitivity; this activity is more potent than that of glycyrrhetinic acid with less toxicity [1]. Besides, bryonolic acid reduces nitric oxide by suppressing inducible nitric oxide synthase expression, indicating anti-inflammatory activity [2]. Interestingly, several studies have reported the ability of bryonolic acid in rat adrenal pheochromocytoma (PC12) cells against *N*-methyl-*D*-aspartate (NMDA)-induced neurotoxicity, indicating it as a candidate neuroprotective agent for cerebral ischemic treatment [3]. Although the sources of bryonolic acid mainly available on cucurbitaceous roots, it was reported only 1.34% *w/w* of dry root from *Cucurbita pepo* [4].

*Trichosanthes* is the largest genus in the Cucurbitaceae family, with more than 100 species in Indochina [5]. This genus has been widely studied because it has a long history of traditional use to treat a variety of diseases. *Trichosanthes cucumerina* L. (TC) is one of the widespread species with various varieties and medicinal potentials [6]. The TC variety cultivated in Thailand is commonly called “Buap Khom” which means bitter gourd because all parts of this variety are incredibly bitter and therefore are not consumed as a vegetable like other cucurbitaceous plants such as cucumbers, watermelons, squashes, and pumpkins. This variety showed unique characteristics, especially its small elliptical fruit [7], which is different from variety cultivated in India, China, and Japan [5,8,9]. TC has traditionally been used as a medicinal plant because of its strong laxative, dandruff treatment, and sinusitis relief properties. Moreover, it was previously reported that its anti-diabetic [10,11], cardioprotective [12], gastroprotective [13], hepatoprotective [14], and cytotoxic [15] activities mainly result from two active triterpenoid compounds, cucurbitacin B and bryonolic acid.

Plant cell cultures are potentially valuable sources for the production of secondary metabolites. These cells, including callus and cell suspension, have been developed during the past decades. They are taken advantage of fast-growing and higher-productive properties for the commercial and industrial productions of plant valuable compounds [16]. Moreover, the application of elicitors is also advantageous for improving desire compounds. Elicitors can induce the production of phytoalexins and stimulating plant defenses to protect the cells leading to the promotion of secondary metabolites; therefore, biotic elicitors have been used in plant cell cultures [17]. Owing to the difficulty of obtaining the triterpenoid productions, there are many evident reports studied on the enhancement of those compounds with elicitor treatments. For example, exogenous methyl jasmonate, a plant signaling molecule, increased soyasaponin production up to six-fold with 100 µM in the licorice cell suspension. Moreover, the expressions of enzymes involved in triterpenoid biosynthesis were also upregulated [18]. The presence of yeast extract, a microorganism-derived elicitor, potentially induced gymnemic acid overproduction in *Gymnema sylvestre* cells reached to six folds [19]. Furthermore, Hu, et al. [20] reported that chitosan treatment was able to induce *Panax ginseng* cultured cells in the accumulation of triterpenoid saponins through upregulating of squalene synthase and squalene epoxidase expressions. Obviously, the key enzymes in the triterpenoid biosynthetic pathway were induced by those types of elicitors; thus, they should be used to optimize the bryonolic acid production.

Because cucurbitacin B and bryonolic acid exhibit numerous activities, many researchers have attempted to improve these compound quantities. Alternative biotechnological strategies, including plant cell cultures, have been studied. Interestingly, TC is one of the major sources for both active triterpenoid compounds. Furthermore, the use of elicitors for improving cucurbitacin B and bryonolic acid production has not been previously reported. In this study, we reported large quantities of bryonolic acid using TC callus and cell suspension. The use of different elicitors (methyl jasmonate (MJ), yeast extract (YE), and chitosan (CH)) at various concentrations were also tested. The cytotoxic activities of these extracts were studied in breast cancer (MCF-7) and osteosarcoma (Saos-2) cell lines. Furthermore, we investigated the wound healing properties of the extracts in fibroblast (Balb/c 3T3) cell lines.

## 2. Results and Discussions

### 2.1. Effect of Explants on Callus and Cell Suspension Induction

**Figure 1:**
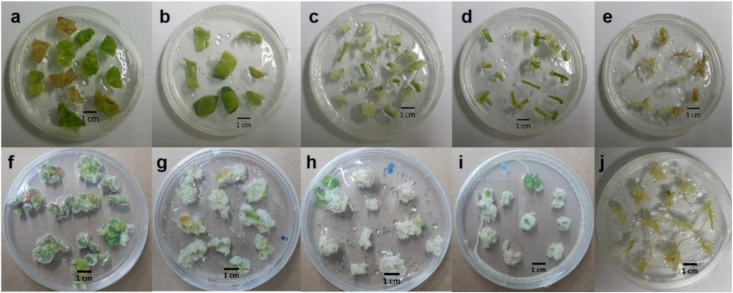
The effect of TC explants from leaves (**a,f**), cotyledons (**b**,**g**), hypocotyls (**c**,**h**), epicotyls (**d**,**i**), and roots (**e**,**j**) on MS media supplemented with 1 mg/L IBA and 1 mg/L BA after subculturing for one week (**a**–**e**) and three weeks (**f**–**j**).

**Figure 2:**
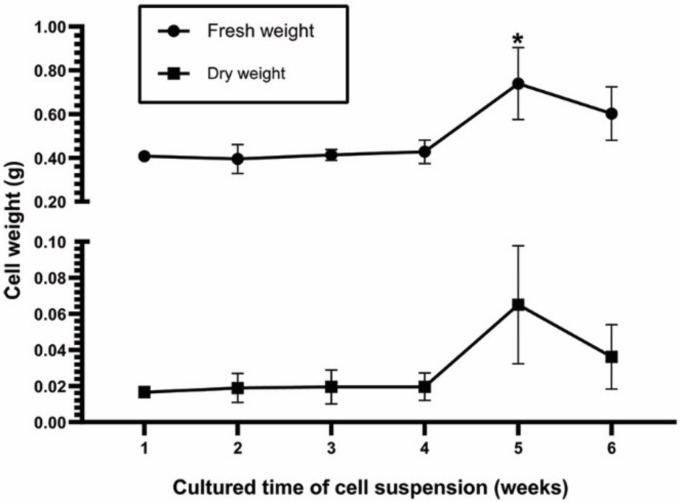
Profile of growth according to fresh and dry weight (g in 30 mL) of TC cell suspensions at the indicated cultured times (week). Data are presented as the mean ± standard deviation from three cell lines. The asterisk indicates significance (* *p*-value < 0.05 by *t*-test from Microsoft Excel 2016).

Explants from the leaves, cotyledons, hypocotyls, epicotyls, and roots of plants grown in vitro were cultured to induce callus formation by culturing them on Murashige and Skoog (MS) plant medium containing 0.5 mg/L indole-3-butyric acid (IBA) and 1 mg/L benzylaminopurine (BAP). From the observation, it can be seen that calli from hypocotyls and epicotyls were white and fragile, meaning that they were appropriate for initiating cell suspensions (Figure 1). Calli from epicotyls were used to establish cell suspensions through culturing in MS liquid medium supplemented with 1 mg/L IBA and 1 mg/L BAP in a shaker at 120 rpm in the dark. The fresh and dry weights of cells were observed for growth rate analyses. The 5-week cell suspension showed the highest fresh and dry weights. The exponential phase of the growth curve was observed from the fourth week to the fifth week, when remarkably increased growth was recorded (Figure 2). Therefore, the four-week cells were used in other experiments, including the study of elicitor effects for enhancing bryonolic acid content.

### 2.2. Effect of Elicitors on Bryonolic Acid Production

**Figure 3:**
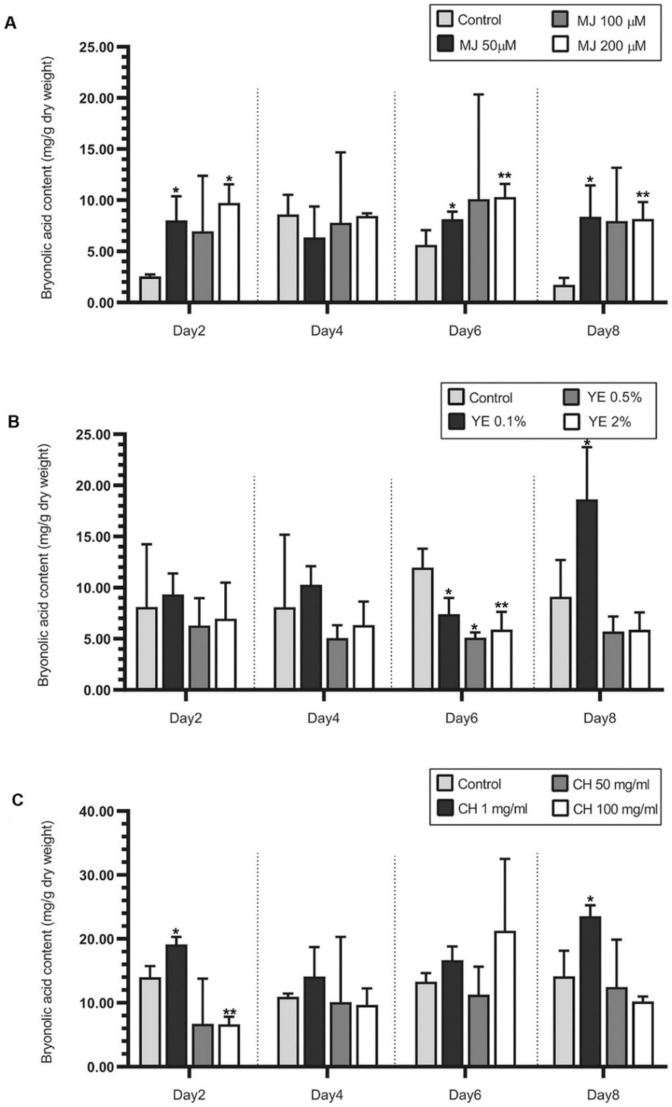
Effects of elicitors on bryonolic acid production in TC cell suspension cultures. (**A**) Methyl jasmonate (50, 100, and 200 µM), (**B**) yeast extract (0.1, 0.5, and 2% *w/v*), and (**C**) chitosan (1, 50, and 100 mg/mL). Elicitors were added to four-week cell suspensions, which were observed after treatment for 2, 4, 6, and 8 days compared with controls (70% ethanol, sterile water, or 1 N acetic acid, respectively). Data are presented as the mean from three independent cell lines, and error bars show the standard deviations. The asterisk indicates significance (* *p*-value < 0.05, ** *p*-value < 0.01 compared with control group by *t*-test from Microsoft Excel 2016).

The effects of elicitors were observed using different concentrations of MJ (50–200 µM), YE (0.1–2% *w*/*v*), and CH (1–100 mg/mL) detected via HPLC analysis. Cell suspensions elicited with 200 µM of MJ for six days or 0.1% *w/v* of YE for eight days were able to produce more bryonolic acid (10.33 ± 1.27 and 18.63 ± 5.10 mg/g dry weight, respectively) than the corresponding control groups (5.63 ± 1.44 and 9.11 ± 3.58 mg/g dry weight, respectively). Comparatively, elicitation with CH at 1 mg/mL provided the best conditions for increasing bryonolic acid levels after treatment for eight days (approximately 23 mg/g dry weight) to levels almost two times higher than in the control groups (Figure 3, Table S6).

It is known that types of elicitors, concentration, and duration of elicitor exposure are important factors affecting the production of secondary metabolites [21]. Plants require different elicited time in response to the different elicitors in any concentrations. From our result, TC cell cultures showed strong responses to MJ elicitation only two days, whereas YE and CH required the delayed responses in the increases of bryonolic acid productions. Sivanandhan, et al. [22] reported that using MJ treatment in *Withania somnifera* hairy roots only 2–8 h potentially increased withanolide A approximately 50-fold. MJ is known as an endogenous elicitor produced after pathogen attack [23]; thus, it probably requires less duration of time than exogenous elicitors produced by pathogens or microorganisms [24].

The use of elicitors in cell suspension culture also offered large quantities of bryonolic acid. This application is appropriate for upscaling the production of interest compounds from small flask to bioreactor. For example, cell suspension cultures of *Withania somnifera*, a medicinal plant known as Ashwagandha or winter cherry, were upscaled in a 5 L bioreactor to obtain greater production of withanolides compared with small flask cultures by approximately 1.5-fold [25].

### 2.3. Metabolic Extraction

In HPLC analysis compared with two standards, cucurbitacin B and bryonolic acid, were detected at 5.8 and 14.2 min, respectively. The extracts from natural roots (TC1), leaves (TC2), stem (TC3), and loofahs (TC4), including cultured cell suspension (TC5), and calli (TC6) were analyzed. Cucurbitacin B was only in TC4 with 4.2 mg/g dry weight. Bryonolic acid was detected in TC1, TC5, and TC6. In our experiments, bryonolic acid contents were measured at approximately 15 mg/g dry weight in TC5 and almost 25 mg/g dry weight by in TC6, whereas the roots were only able to produce this compound at approximately 2 mg/g dry weight (Table S5). Although alternative methods have previously been developed for bryonolic acid isolation, the maximum content was only 13.4 mg/g dry weight by root cultures throughout *C. pepo* with specific conditions [4]. Through the plant cell cultures and hairy roots, bryonolic acid was produced only 10–13 mg/g dry weight [26,27]. Therefore, TC is a valuable novel source in bryonolic acid purification.

### 2.4. Biological Activities

**Figure 4:**
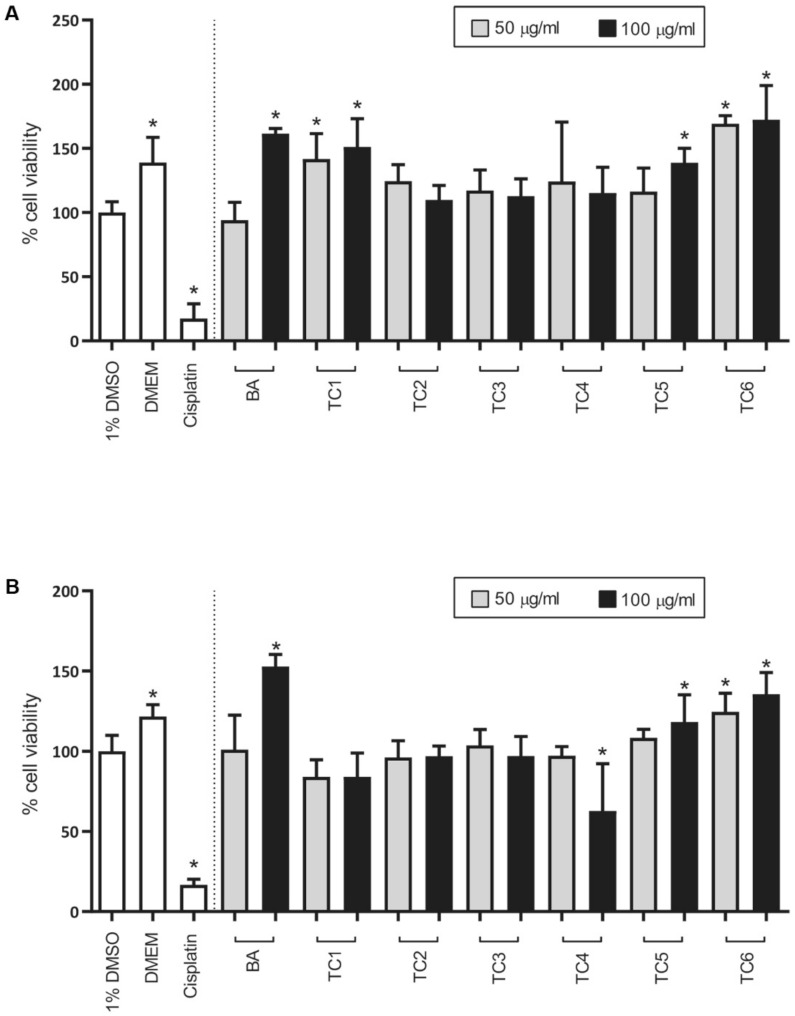
Effect of two concentrations (50 and 100 µg/mL) of MeOH extracts (TC1: root, TC2: leaf, TC3: stem, TC4: loofah, TC5: cell suspension and TC6: callus) and bryonolic acid (BA) compared with 1% *v/v* DMSO in the (**A**) MCF-7 and (**B**) Saos-2 cell lines. DMEM and cisplatin were used as negative and positive controls, respectively. The bar graph and error bars represent the mean and standard deviation from the experiments, respectively. The asterisk indicates significance (* *p*-value < 0.05 compared with the 1% *v/v* DMSO group).

In the presence of the six TC extracts (TC1–TC6), including the bryonolic acid standard, none of the extracts at a concentration of 50 or 100 µg/mL showed toxicity in either the Saos-2 or MCF-7 cell lines. Conversely, the TC1, TC5, and TC6 extracts resulted in a significantly higher percentage of cell viability in MCF-7 cells by 1.4–1.7 times than in the control treatment group (1% *v/v* DMSO) (Figure 4A). Moreover, the TC5 and TC6 extracts increased the percentage of cell viability in Saos-2 cells by 1.1–1.3 times compared with 1% *v/v* DMSO treatment (Figure 4B). Similarly, bryonolic acid at 100 µg/mL resulted in 1.6 times higher cell viability of both cell lines compared with 1% *v/v* DMSO. Previously, two antifungal agents, fludioxinil and fenhexamid, which have structurally similar to 17β-estradiol, were reported with the capacity of the induction in MCF-cell proliferations through estrogen receptor by 1.2 to 1.5 times and 2.5 times, respectively, compared to control (0.1% *v/v* DMSO) treatment [28,29]. Although bryonolic acid has been shown to exert a neuroprotective effect against NMDA in rat adrenal pheochromocytoma (PC12) cell lines [3], it also showed cytotoxic activity against various other cell lines, such as lung cancer cells (A549) and breast cancer cells (MDA-MB435, SKBR3, MCF-7, and T47D) [15]. From these reported findings, it would conclude that bryonolic acid exhibits cell-type specific properties; however, additional experiments are needed to confirm this hypothesis. Because of the presence of bryonolic acid peaks in TC1, TC5, and TC6 (Figure S3), we assumed that bryonolic acid may have a synergistic effect with other compounds in the extracts increasing MCF-7 cell viability. These observations suggested that TC1, TC5, TC6, and bryonolic acid may induce the proliferations of the treated cell lines. It is well known that cell proliferation is one of the main characteristics of the wound-healing process [30,31]. Based on this assumption, we further examined the wound-healing properties of these three extracts with the hypothesis of cytoprotection.

**Figure 5:**
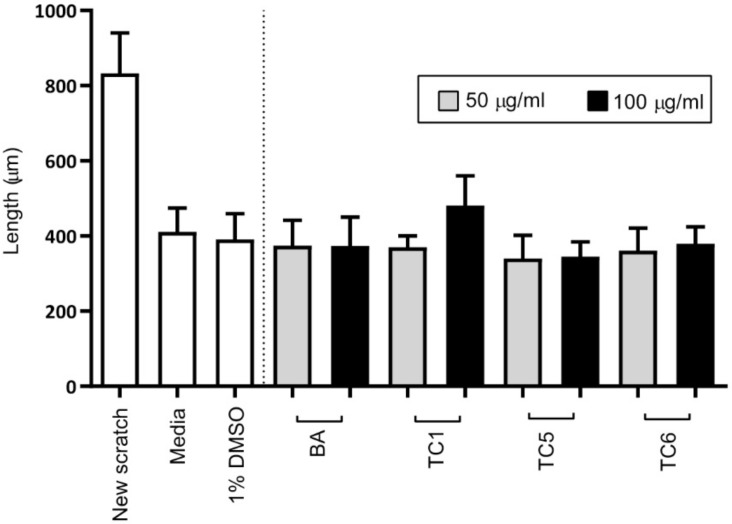
Wound healing assay in Balb/c 3T3 cells treated with two concentrations (50 and 100 µg/mL) of MeOH extracts (TC1: root, TC5: cell suspension and TC6: callus) and bryonolic acid (BA) compared with 1% *v/v* DMSO treatment. The bar graph and error bars represent the mean and standard deviation of the length (µm) from the experiments.

A wound-healing assay was performed using two-dimensional confluent monolayer Balb/c 3T3 cells. The length or wound width, which represents the migration rate of cells, was evaluated after 14 h of exposure to the extracts. Cells that were treated with all extracts and bryonolic acid showed slightly narrower wound widths than the cells treated with 1% *v/v* DMSO, except for those treated with TC1 at the concentration of 100 µg/mL. Among all groups tested, the extract from cell suspensions (TC5) resulted in the narrowest wound with no significant difference (Figure 5). Although our extracts led to the increased cell viability, they could not clearly represent the wound-healing effect. As mentioned before, wound-healing effect can be performed through cell proliferation. Many reports suggested that this activity was also able to process by other mechanisms. It was presented that leaf extract of *Chromolaena odorata* exerted wound-healing property by the increase of heme oxygenase-1 (HO-1) [32]. Moreover, cell migration was also a key role in this activity [33]. Accordingly, further validated study on cell proliferation would potentially help in the cytoprotective hypothesis.

## 3. Materials and Methods

### 3.1. Chemicals

Indole-3-butyric acid (IBA) and benzylaminopurine (BAP) were purchased from Tokyo Chemical Industry Co., Ltd (Tokyo, Japan). Methyl jasmonate (95%) and chitosan (from shrimp shells, ≥75%) were purchased from Sigma-Aldrich, St. Louis, USA. Granulated yeast extract was purchased from Merck, Darmstadt, Germany. Dulbecco’s modified Eagle’s medium (DMEM) powder (low glucose), Dulbecco’s phosphate-buffered saline (DPBS) without calcium chloride and magnesium chloride, fetal bovine serum (FBS), PrestoBlue^®^ cell viability reagent, and 0.25% trypsin-EDTA were purchased from Life Technologies Corporation, CA, USA. Penicillin-streptomycin 100X was obtained from Merck KGaA, Darmstadt, Germany. Cisplatin was purchased from SuZhou Rovathin Foreign Trade Co., Ltd (Jiangsu, China). Dimethyl sulfoxide (DMSO) (plant cell culture tested) was purchased from Sigma-Aldrich, St. Louis, USA. Cucurbitacin B and bryonolic acid were provided by Prof. Dr. Weena Jiratchariyakul and were previously isolated from TC fruit juice and roots and confirmed [15].

### 3.2. Plant Materials

The natural plant materials were kindly obtained in 2015 (Nakhon Pathom province, Thailand) from Prof. Dr. Weena Jiratchariyakul, Faculty of Pharmacy, Mahidol University, Thailand (BKF no. 70279). The dried roots, leaves, stems, and loofahs were powdered and extracted for HPLC analysis. The seeds were sterilized with 70% *v/v* ethanol for 1 min and 0.5% *v/v* sodium hypochlorite for 5 min. Then, they were washed with sterile water three times. The sterile seeds were soaked in sterile water for two days before culturing on MS hormone-free medium. The pH of the medium was adjusted to 5.7 ± 0.1 before autoclaving at 121 °C for 15 min. The cultures were maintained at 25 ± 2 °C in the dark.

### 3.3. Callus Induction

After seed germination, the seedlings were cut and cultured on MS medium supplemented with 0.5 mg/L IBA and 1 mg/L BAP for inducing callus [34]. The explants from leaves, cotyledons, hypocotyls, epicotyls, and roots (*n* = 25) were observed under conditions of temperature maintained at 25 ± 2 °C and a 16 h photoperiod (light intensity at 2000 lux).

### 3.4. Cell Suspension Initiation

The calli were used for preparing cell suspension cultures. Approximately 0.5 g of calli were cultured in 125 mL Erlenmeyer flasks containing 30 mL of MS liquid medium supplemented with 1 mg/L of IBA and 1 mg/L of BAP in a shaker at 120 rpm in the dark to establish cell suspensions [34]. The cell suspension cultures were routinely subcultured every 30 days. The cell suspensions were harvested from the first week until the sixth week for a growth rate study. The fresh and dry weights (g in 30 mL) of these cell suspensions were recorded.

### 3.5. Effect of Elicitors on Bryonolic Acid Accumulation

Different concentrations of MJ (50, 100, and 200 µM), CH (1, 50, and 100 mg/L), and YE (0.1, 0.5, and 2% *w/v*) were added to 4-week cell suspensions cultured in 30 mL of MS liquid medium containing 1 mg/mL IBA and 1 mg/mL BAP. The treated groups of cell suspensions were observed after elicitation for 2, 4, 6, and 8 days compared with the control groups for MJ, CH, and YE administered 70% *v/v* ethanol, 0.1 N acetic acid, or water, respectively.

### 3.6. Active Compound Analysis Using High-Performance Liquid Chromatography (HPLC)

Approximately 100 mg of each dried sample was extracted with 1 mL of methanol (MeOH) containing 200 µg/mL of progesterone as an internal standard. Each extract was filtered through a 0.45 µm syringe filter before analysis by HPLC (LC-20A, Shimadzu) with a BDS HYPERSIL C18 column (Dim. 250 × 4.6 mm, particle size 5 µm) with an injection volume of 10 µL. The mobile phases were MeOH and water: 0–2.5 min (70% *v/v* MeOH), 2.51–10 min (100% *v/v* MeOH) and 10.01–19.00 min (70% *v/v* MeOH), at a flow rate of 1 mL/min. The extracts were detected at 210 nm compared with cucurbitacin B and bryonolic acid standards for quantification based on standard calibration curves. These two standards were reconfirmed via high-resolution ESI-MS (Figures S1 and S2). The HPLC conditions were validated. Linear regression equation, correlation coefficient (r^2^), and the limit of quantitation (LOQ) were determined through five concentrations of cucurbitacin B and bryonolic acid (500, 250, 125, 100, and 50 µg/mL). The precision was confirmed by the relative standard deviation (%RSD). The accuracy was performed based on %recovery with three-level concentrations of both compounds (400, 125, and 75 µg/mL) (Tables S1–S3).

### 3.7. Cytotoxicity Test

Saos-2 osteosarcoma (ATCC number HTB-85) and MCF-7 breast cancer (ATCC number HTB-22) cells were cultured in DMEM supplemented with 10% fetal bovine serum and penicillin-streptomycin (100 units/mL) at 37 °C under a 5% CO_2_ humidified atmosphere. The medium was changed every 2–3 days. After reaching 70–80% confluency, the cells were detached with 0.25% trypsin-EDTA for subculture.

Stock solutions (10 mg/mL) of MeOH extracts from the roots (TC1), leaves (TC2), stems (TC3), loofahs (TC4), cell suspensions (TC5), and calli (TC6) were prepared by dissolving the dried methanolic extracts in DMSO. Bryonolic acid was also tested in this experiment for comparison. Two concentrations of TC extracts (50 and 100 µg/mL) were prepared via the following protocol. A 100 µg/mL extract was prepared from the stock solution by a 100-fold dilution in DMEM. Subsequently, this solution was diluted two-fold in DMEM containing 1% *v/v* DMSO to yield 50 µg/mL of extract. The final concentration of DMSO in all samples was set at a constant 1% *v/v*.

The toxicity of the extract was tested in the Saos-2 and MCF-7 cell lines. Cells (100 µL) were seeded in 96-well plates at cell densities of 2000 and 4000 cells per well for Saos-2 and MCF-7 cells, respectively. After incubation under a 5% CO_2_ humidified atmosphere at 37 °C for 24 h, the medium was discarded, and 100 µL of the sample was added to each well. After 48 h of incubation, 10 µL of PrestoBlue^®^ reagent was added, followed by incubation for another 50 min. Absorbance values were recorded at 570 and 600 nm as the measurement and reference wavelengths, respectively, in a Tecan Infinite^®^200 microplate reader (NanoQuant, Männedorf, Switzerland). Cells incubated with DMEM containing 1% *v/v* DMSO and cisplatin at 10 µg/mL were used as negative and positive controls. The measurement was performed in triplicate on three different days.

### 3.8. Wound-Healing Test

Mouse fibroblast cell lines (Balb/c 3T3 close A31, ATCC^®^ CCL-163TM from American Type Culture Collection (ATCC), MD, USA) were maintained in DMEM. The media were supplemented with 10% bovine calf serum (Donor bovine serum with iron Gibco^®^, Life Technologies Corporation, CA, USA) and 1% penicillin/streptomycin. Cells were incubated at 37 °C under 5% CO_2_. The medium was replenished every three days. Subculturing was performed before the cells were completely confluent.

To evaluate the cell migration of a confluent monolayer, an in vitro wound-healing assay was conducted by scratching a two-dimensional confluent culture [35]. Balb/c 3T3 cells were seeded at a concentration of 0.3 × 106 cells per well in 12-well plates one day prior to the experiment. Then, scratches were made using 200 µL micropipette tips (three scratches per well), followed by gently washing off the cell debris with PBS. Cells were replenished with fresh medium containing the extracts from TC1, TC5, and TC6 at concentrations of 50 and 100 µg/mL. Since the TC extracts were dissolved in DMSO, 1% *v/v* DMSO served as a control. After 14 h of incubation, the cells were washed with PBS and then fixed with 4% paraformaldehyde. Images of the scratched area were captured using a CKX53 inverted microscope (Olympus^®^, Shinjuku, Tokyo) at a magnification of 10×. For each concentration of TC extract, nine scratches were made in three 12-well plates, and five images per scratch were obtained. ImageJ (ImageJ 1.50i, National Institutes of Health, USA) was used to measure the wound width (µm).

### 3.9. Statistical Analysis

The experimental results are reported as the mean ± standard deviation. The cytotoxicity data were statistically analyzed with GraphPad Prism^®^ version 7.00 for Windows (GraphPad Software, La Jolla, CA, USA, www.graphpad.com). Statistical significance was determined using one-way ANOVA and Dunnett’s multiple comparisons test (*p*-value < 0.05).

## 4. Conclusions

In this study, plant cell cultures and the effects of elicitors on bryonolic acid were investigated. The results of the study showed notably amounts of bryonolic acid compared with those of the TC plant parts from nature by way of TC callus and cell suspension cultures. The presence of chitosan in cell suspension culture remarkably increases the production of bryonolic acid compared with the control group. Besides, the cytotoxic activities of the extracts from calli, cell suspensions and roots with high bryonolic acid contents were associated with a significantly greater percentage of cell viability than 1% *v/v* DMSO treatment. Bryonolic acid has an interesting pleiotropic profile of biological activities, including immunomodulatory, anti-inflammatory, cytoprotective, and cytotoxicity effects, as mentioned above. Thus, bryonolic acid is a promising candidate for further investigation to explore mechanisms underlying biological activities and for the development of new drugs in the future. This finding provides a novel plant source and an alternative method through plant cell culture techniques to improve the production of bryonolic acid for further needs.

## Supplementary Materials

The following are available online at https://www.mdpi.com/2223-7747/9/6/709/s1. Table S1: The effect of explants from *T. cucumerina* L. in the induction of calli; Table S2: Calibration data of cucurbitacin B and bryonolic acid; Table S3: Intra-day and Inter-day precisions of HPLC method; Table S4: Accuracy of HPLC method; Table S5: Averages of bryonolic acid contents from MeOH extracts of root, leaf, stem, loofah, cell suspension, and callus; Table S6: Averages of bryonolic acid contents in MeOH extracts from TC cell suspension cultures after treated with elicitors; Figure S1: *T. cucumerina* L. (Buapkhom); Figure S2 and S3: High-resolution ESI-MS of cucurbitacin B and bryonolic acid standard; Figure S4: HPLC chromatogram of MeOH extracts.
