# Supplementary material for: Enhanced Production of Bryonolic Acid in Trichosanthes cucumerina L. (Thai Cultivar) Cell Cultures by Elicitors and Their Biological Activities
Source: Plants (Basel). 2020 Jun 2;9(6):709. doi: 10.3390/plants9060709 (PMC7356870; doi:10.3390/plants9060709)
Supplement: Supplementary file 1 [file plants-09-00709-s001.pdf]

Supplementary material

Table 1. The effect of explants from *T. cucumerina* L. in the induction of calli on MS medium supplemented with 0.5 mg/l IBA and 1 mg/l BA.

Explants	No. of Explants Cultures	Percent of Explant Forming Callus	Morphogenic Responsive of Explant
Leaves	25	100	Green, condensed callus
Cotyledons	25	100	Green or white, condensed callus
Hypocotyls	25	100	White, fragile callus
Epicotyls	25	100	White, fragile callus
Roots	25	0	-

Table 2. Calibration data of cucurbitacin B and bryonolic acid, including linear regression equation, correlation coefficient (r^2), the limit of quantitation (LOQ), and range.

Compound	Linear regression equation	r^2	LOQ (μg/ml)	Range (μg/ml)
Cucurbitacin B	$y = 0.0054x - 0.0024$	0.9990	22	50-500
Bryonolic acid	$y = 0.0029x - 0.0075$	0.9975	35	50-500

Table 3. Intra-day and Inter-day precisions of HPLC method; results are reported on mean ± standard deviation of % the relative standard deviation (%RSD).#

Compound	Intra-day	Inter-day
Cucurbitacin B	5.04 ± 1.13	3.61 ± 0.36
Bryonolic acid	5.38 ± 1.99	4.14 ± 0.50

Table 4. Accuracy of HPLC method; results are based on %recovery.

Compound	Concentration (μg/ml)	Found (μg/ml)	%recovery
Cucurbitacin B	Low (75)	75.07 ± 3.68	100.09 ± 4.91
	Medium (125)	119.12 ± 7.42	95.30 ± 5.94
	High (400)	373.07 ± 14.90	93.27 ± 3.72
Bryonolic acid	Low (75)	75.55 ± 4.82	98.07 ± 6.43
	Medium (125)	118.60 ± 7.70	94.88 ± 6.16
	High (400)	373.47 ± 11.50	93.37 ± 2.87

Table 5. Averages of bryonolic acid contents from MeOH extracts of root, leaf, stem, loofah, cell suspension and callus. Results are reported as mean ± standard deviation. (Linear regression equation of Cucurbitacin B and bryonolic acid: $y = 0.0058x - 0.0734$ ($r^2 = 0.9999$), $y = 0.0027x + 0.1105$ ($r^2 = 0.9881$), respectively).

Sample	Content (mg/g dry weight)	
	Cucurbitacin B	Bryonolic acid
Root (TC1)	-	2.47 ± 0.58
Leaf (TC2)	-	-
Stem (TC3)	-	-
Loofah (TC4)	4.20 ± 0.35	-
Cell suspension (TC5)	-	15.69 ± 0.78
Callus (TC6)	-	24.65 ± 1.97

Table 6. Averages of bryonolic acid contents in MeOH extracts from TC cell suspension cultures after treated with elicitors including methyl jasmonate (50 – 200 µM), chitosan (1 – 100 mg/ml), and yeast extract (0.1 – 2%) for 2, 4, 6, and 8 days compared with controls (70% ethanol, 1N acetic acid, and sterile water, respectively). Results are reported as mean ± standard deviation. The asterisk indicates significance (**p* < 0.05, ***p* < 0.01 compared with control group).

**Elicitors**	**Concentration**	**Average of bryonolic acid content (mg/g dry weight)**			
		Day2	Day4	Day6	Day 8
Methyl Jasmonate (MJ)	Control	2.55 ± 0.19	8.62 ± 1.91	5.63 ± 1.44	1.73 ± 0.68
	50 µM	8.03 ± 2.35 *	6.37 ± 3.01	8.17 ± 0.72 *	8.38 ± 3.06 *
	100 µM	6.97 ± 5.42	7.80 ± 6.88	10.11 ± 10.24	7.97 ± 5.21
	200 µM	9.74 ± 1.82 *	8.46 ± 0.26	**10.33 ± 1.27 ****	8.18 ± 1.64 **
Chitosan (CH)	Control	14.00 ± 1.74	10.96 ± 0.50	13.31 ± 1.33	14.14 ± 4.00
	1 mg/ml	19.16 ± 1.14 *	14.12 ± 4.59	16.69 ± 2.15	**23.56 ± 1.68**
	50 mg/ml	6.72 ± 7.07	10.12 ± 10.21	11.28 ± 4.36	12.49 ± 7.38
	100 mg/ml	6.65 ± 1.17 **	9.69 ± 2.56	21.27 ± 11.23	10.23 ± 0.74
Yeast extract (YE)	Control	8.11 ± 6.14	8.10 ± 7.07	11.97 ± 1.84	9.11 ± 3.58
	0.1%	9.35 ± 2.05	10.28 ± 1.81	7.42 ± 1.58 *	**18.63 ± 5.10 ***
	0.5%	6.31 ± 2.66	5.07 ± 1.25	5.12 ± 0.50 *	5.71 ± 1.47
	2%	6.98 ± 3.51	6.34 ± 2.29	5.91 ± 1.71 **	5.89 ± 1.69

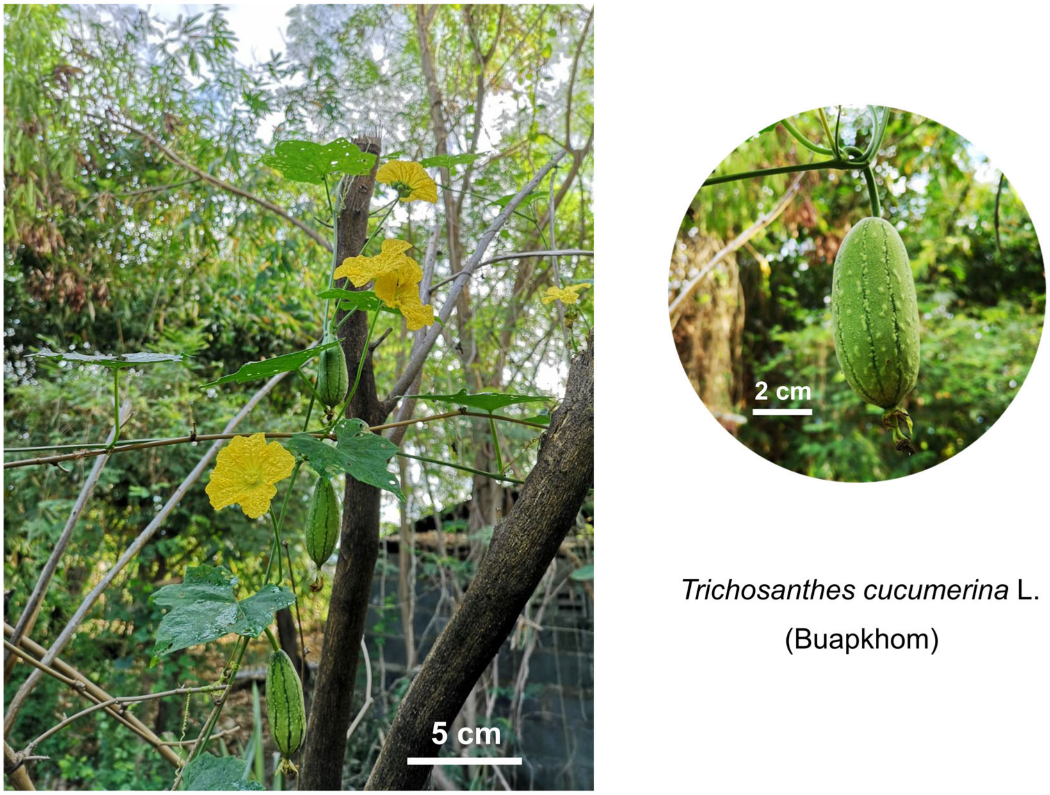

Figure S1. *Trichosanthes cucumerina* L. (Buapkhom).

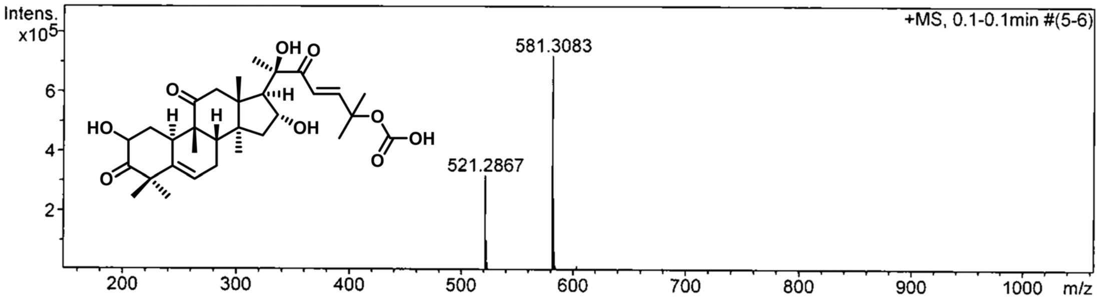

Figure S2. High-resolution ESI-MS of cucurbitacin B standard (m/z 581.3083).

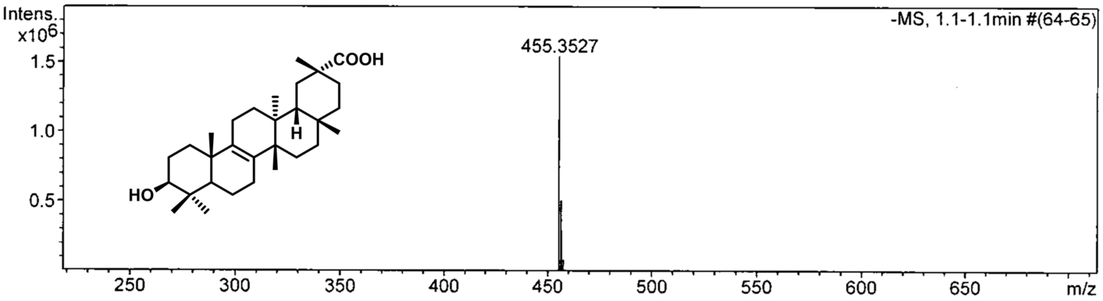

Figure S3. High resolution ESI-MS of bryonolic acid (m/z 455.3527).

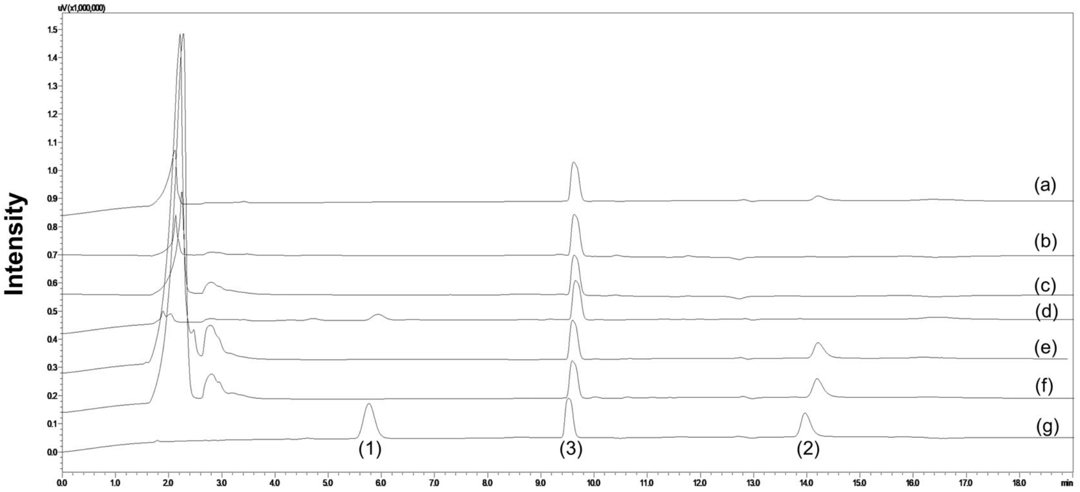

Figure S4. HPLC chromatogram of MeOH extracts (a) root (TC1), (b) leaf (TC2), (c) stem (TC3), (d) loofah (TC4), (e) cell suspension (TC5), and (f) callus (TC6) compared with two standards (g) as 250 μg/ml of cucurbitacin B (1) and 250 μg/ml of bryonolic acid (2). The extracts were detected at 210 nm and supplemented with 200 μg/ml of progesterone (3) as an internal standard.
